# Colonic Angiosarcoma 16 Years After Pelvic Radiotherapy for Cervical Cancer: A Case Report

**DOI:** 10.7759/cureus.117323

**Published:** 2026-10-01

**Authors:** Ayoub Bouziane, Btissam Essâdi, Chaimae Bekhakh, Amal Bennani, Ouiam Elmqaddam, Hajar Koulali, Zahi Ismaili, Ghizlane Kharrasse, Abdelkrim Zazour

**Affiliations:** 1 Gastroenterology and Hepatology, Mohammed VI University Hospital, Oujda, MAR; 2 Pathology and Digestive Oncology, Faculty of Medicine and Pharmacy of Oujda, Mohammed First University Oujda, Oujda, MAR; 3 Pathology, Faculty of Medicine and Pharmacy of Oujda, Mohammed First University Oujda, Oujda, MAR

**Keywords:** angiosarcoma, case report, colonic neoplasms, pelvic radiotherapy, radiation-associated sarcoma

## Abstract

Angiosarcomas are rare and aggressive malignant neoplasms of vascular or lymphatic endothelial origin, accounting for approximately 2% of soft-tissue sarcomas. They occur predominantly in the skin and superficial soft tissues, particularly in the head and neck region and the breast, whereas gastrointestinal involvement is exceptionally rare. Previous radiotherapy is a well-established risk factor for secondary angiosarcoma. However, radiation-associated gastrointestinal angiosarcomas are extremely uncommon and have been reported predominantly in the small bowel.

We report the case of a 55-year-old woman who presented with abdominal pain, hemorrhagic ascites, intermittent rectal bleeding, severe anemia, anorexia, and weight loss 16 years after pelvic radiotherapy and cisplatin-based chemotherapy for cervical squamous cell carcinoma. Computed tomography revealed circumferential thickening of the cecum and right colon with abundant hyperdense ascites. Colonoscopy showed an obstructing, ulcerated, and contact-bleeding mass involving the cecum and right colon. Histopathological examination of endoscopic biopsies revealed a poorly differentiated malignant vascular neoplasm. Immunohistochemical staining was positive for cluster of differentiation (CD)31 and CD34 and negative for cytokeratin, CD3, CD20, and Human Herpesvirus (HHV)8, with a Ki-67 proliferation index of 40%, supporting the diagnosis of colonic angiosarcoma. Surgical resection was considered; however, the patient developed a massive bilateral pulmonary embolism and died seven days after admission.

Given the prolonged latency and the histological distinction from the primary cervical carcinoma, a possible radiation-associated etiology was considered. Nevertheless, the original radiotherapy plan was unavailable, preventing definitive confirmation that the tumor arose within the irradiated field. This case highlights the diagnostic difficulty, aggressive clinical course, and poor prognosis of colonic angiosarcoma following pelvic radiotherapy.

## Introduction

Angiosarcoma is a rare and highly aggressive malignant tumor arising from vascular or lymphatic endothelial cells, accounting for approximately 2% of soft-tissue sarcomas. It most commonly involves the skin and superficial soft tissues of the head and neck, as well as the breast, whereas visceral involvement is uncommon. Primary gastrointestinal angiosarcoma is exceptionally rare, and colorectal involvement has been described predominantly through isolated case reports and small literature reviews [1-3].

Angiosarcomas may occur as primary tumors or develop secondary to predisposing factors. The best-recognized risk factors include chronic lymphedema, previous radiotherapy, exposure to certain chemical carcinogens, and rare familial cancer syndromes. Radiotherapy is among the most clearly established risk factors for secondary angiosarcoma. Radiation-associated angiosarcomas have been reported mainly in the breast and superficial soft tissues, whereas gastrointestinal involvement remains exceptional. Among reported gastrointestinal cases, the small bowel is the most frequently affected site, while radiation-associated colorectal angiosarcoma is particularly rare [4,5].

Because of its rarity and nonspecific clinical presentation, colorectal angiosarcoma may mimic more common colorectal neoplasms, particularly adenocarcinoma, and its diagnosis relies on histopathological and immunohistochemical examination. We report a rapidly fatal case of colonic angiosarcoma in a 55-year-old woman, diagnosed 16 years after pelvic radiotherapy for cervical squamous cell carcinoma. We also discuss the diagnostic criteria relevant to a possible radiation-associated etiology and review the relevant literature.

## Case presentation

A 55-year-old woman presented to the emergency department with abdominal pain, progressive abdominal distension, intermittent rectal bleeding, loss of appetite, weight loss, and deterioration in her general condition. She had a history of squamous cell carcinoma of the uterine cervix diagnosed 16 years earlier, for which she underwent total hysterectomy with bilateral salpingo-oophorectomy, followed by adjuvant pelvic radiotherapy delivered in 24 fractions and cisplatin-based chemotherapy. The total radiation dose, detailed treatment plan, and irradiation fields were unavailable. She reported no other relevant personal or family history of malignancy.

On physical examination, the patient was conscious and normotensive but markedly pale and tachycardic, with a heart rate of 120 beats/min. Abdominal examination revealed marked distension with clinical signs of abundant ascites. There was no peripheral lymphadenopathy, hepatomegaly, or splenomegaly. Digital rectal examination revealed no palpable lesion or visible blood.

Laboratory investigations on admission revealed severe anemia, with a hemoglobin level of 6.3 g/dL, as well as hypoalbuminemia. Renal function was preserved, and the C-reactive protein level was 6 mg/L. Serum carcinoembryonic antigen and carbohydrate antigen 19-9 levels were within the reference ranges, whereas the cancer antigen (CA)-125 level was elevated (Table 1).

**Table 1:** Laboratory findings CEA: carcinoembryonic antigen; CRP: C-reactive protein; CA 19-9: carbohydrate antigen 19-9; CA-125: cancer antigen 125.

Laboratory parameter	Patient value	Reference range
Hemoglobin	6.3 g/dL	12–16 g/dL
C-reactive protein (CRP)	6 mg/L	0–5 mg/L
Serum albumin	28 g/L	35–50 g/L
Serum creatinine	6.2 mg/L	5.7–11.1 mg/L
Carcinoembryonic antigen (CEA)	1 ng/mL	<5 ng/mL
CA 19-9	4 U/mL	<37 U/mL
CA-125	120 U/mL	<35 U/mL

HIV serology was negative. Large-volume abdominal paracentesis yielded hemorrhagic ascitic fluid with a protein concentration of 45 g/L. Cytological examination of the ascitic fluid showed no malignant cells.

Because of the hemorrhagic nature of the ascites, contrast-enhanced computed tomography of the abdomen and pelvis was performed. It showed symmetrical circumferential wall thickening involving the cecum and right colon (Figures 1A, 1B), associated with abundant hyperdense ascites (Figure 1C) and no evidence of active contrast extravasation.

**Figure 1:**
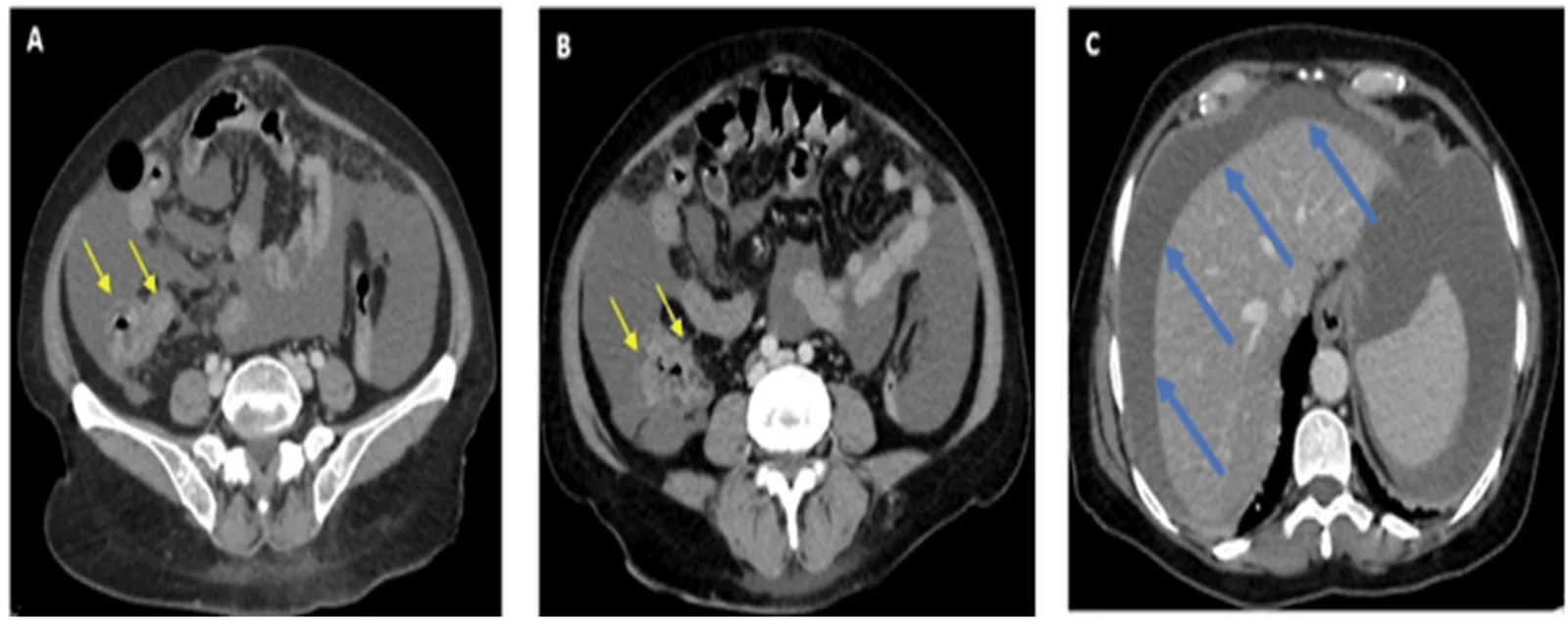
Contrast-enhanced CT findings (A, B) Axial contrast-enhanced CT images showing circumferential wall thickening of the cecum and right colon (yellow arrows); (C) Axial contrast-enhanced CT image showing abundant ascites (blue arrows).

No focal hepatic or splenic lesions or enlarged deep lymph nodes were identified. No aortic or mesenteric vascular thrombosis was detected.

During hospitalization, the patient received three units of packed red blood cells. Colonoscopy revealed a circumferential, ulcerated, stenosing mass involving the cecum and right colon. The lesion bled on contact and caused complete luminal obstruction. Endoscopic features consistent with chronic radiation proctitis were also observed (Figure 2).

**Figure 2:**
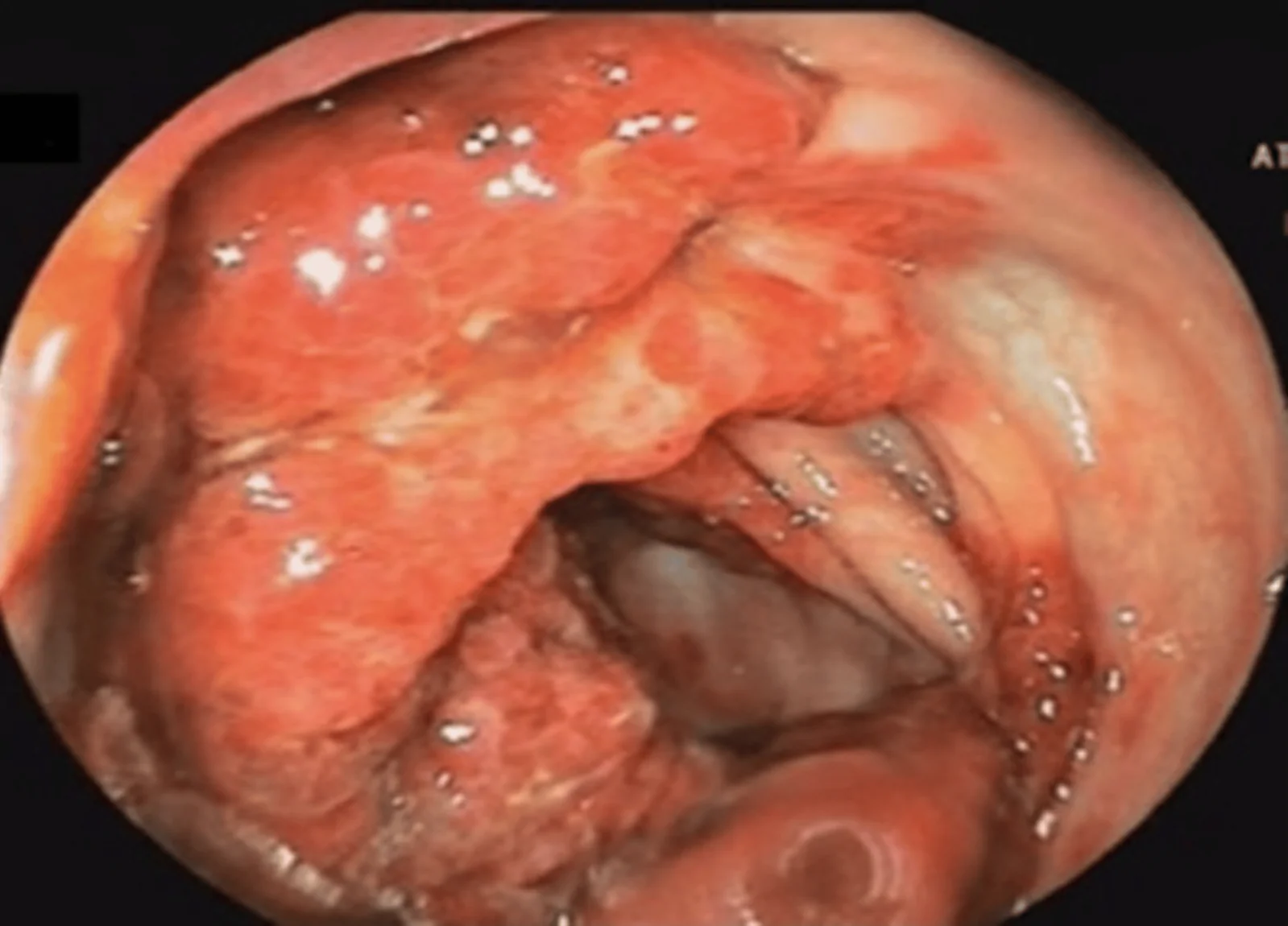
Colonoscopy revealed a stenosing, hyperemic, hemorrhagic and friable circumferential mass of the right colon and cecum

Multiple endoscopic biopsies were obtained from the colonic mass. Histopathological examination revealed a poorly differentiated malignant vascular neoplasm composed of irregular vascular channels and solid sheets of atypical endothelial cells. The tumor cells exhibited enlarged, hyperchromatic nuclei with irregular contours and scant cytoplasm (Figure 3).

**Figure 3:**
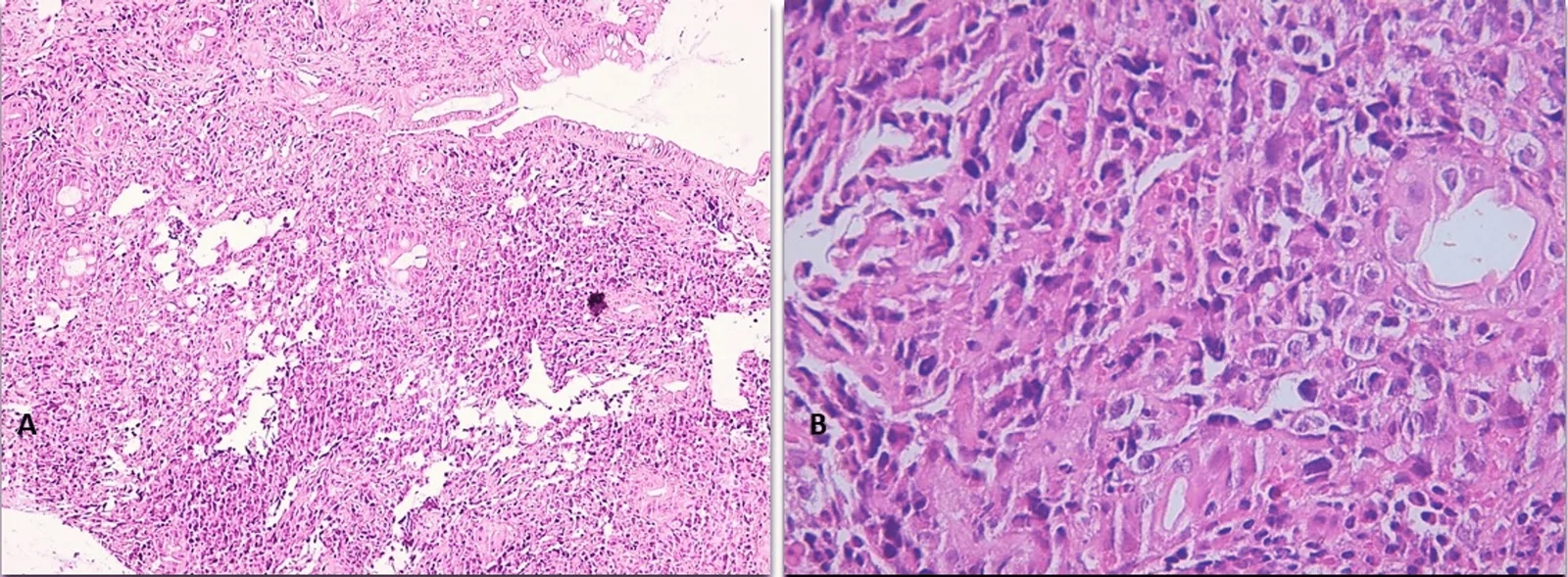
A poorly differentiated angiosarcoma of the colon involving lamina propria and showing cords and sheets of atypical endothelial cells A (Hematoxylin and Eosin or H&E x10); B (H&E x20).

Immunohistochemical analysis showed diffuse tumor-cell expression of the endothelial markers cluster of differentiation (CD)31 and CD34. The tumor cells were negative for cytokeratin, CD3, CD20, and human herpesvirus 8 (HHV8). The Ki-67 proliferation index was approximately 40% (Figure 4).

**Figure 4:**
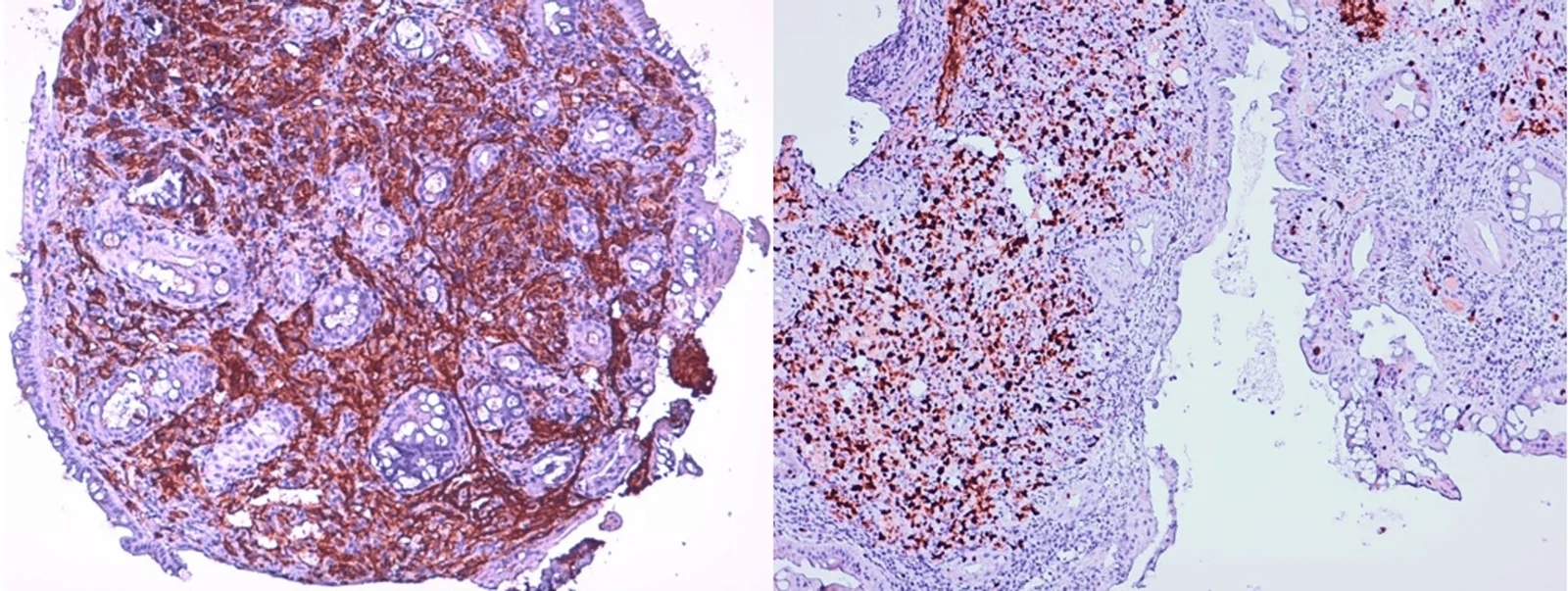
Immunohistochemistry showed diffuse anti-CD31 marking and a significant elevation in Ki-67 expression

The morphological and immunohistochemical findings supported the diagnosis of a poorly differentiated colonic angiosarcoma.

The case was discussed at a multidisciplinary tumor board, and surgical resection was considered because of the obstructing and bleeding nature of the tumor. However, before surgery could be performed, the patient developed a massive bilateral pulmonary embolism, confirmed by computed tomography pulmonary angiography, and died seven days after admission.

## Discussion

Angiosarcoma is a rare and highly aggressive malignant neoplasm of vascular or lymphatic endothelial origin, accounting for approximately 2% of soft-tissue sarcomas. It predominantly affects the skin and superficial soft tissues, particularly in the head and neck region and the breast, whereas primary visceral angiosarcoma is uncommon. Within the abdomen, the liver, spleen, adrenal glands, and ovaries are the most frequently reported primary sites. Gastrointestinal angiosarcoma is exceptionally rare and is associated with an aggressive clinical course and poor prognosis [1,4,5].

In a systematic review of 110 patients with primary gastrointestinal angiosarcoma, Schizas et al. reported that the small intestine was the most frequently involved site (44.5%), followed by the large intestine (35.5%) [5]. Only a minority of the reported gastrointestinal angiosarcomas were associated with previous radiotherapy, emphasizing the exceptional nature of radiation-associated colorectal involvement. However, the exact number of published cases varies among reviews because of differences in the inclusion of primary, metastatic, multifocal, and radiation-associated tumors. Li et al. reviewed 66 cases of small-bowel angiosarcoma published between 1970 and 2017, including a subgroup associated with previous radiotherapy [6]. Among these radiation-associated cases, most patients were women who had previously received pelvic radiotherapy for gynecological malignancies. The reported latency between radiotherapy and angiosarcoma diagnosis ranged from three to 20 years, with a mean interval of approximately 10 years. The terminal ileum was the most frequently affected segment, and metastatic disease, particularly involving the liver, lungs, or peritoneum, was common at diagnosis [6]. These epidemiological findings relate primarily to small-bowel angiosarcoma and should not be directly extrapolated to colorectal disease.

Radiation-associated colorectal angiosarcoma remains particularly uncommon and has been described mainly in isolated case reports. Other recognized risk factors for angiosarcoma include chronic lymphedema, exposure to vinyl chloride or thorium dioxide, and rare familial cancer-predisposition syndromes [4,7,8]. Our patient had undergone pelvic radiotherapy 16 years before the diagnosis of colonic angiosarcoma, a latency consistent with previously reported radiation-associated cases. Nevertheless, because the original radiotherapy plan was unavailable, the precise relationship between the tumor site and the irradiated field could not be definitively established. Radiation-associated sarcoma is commonly assessed using the modified Cahan criteria, which include: (1) a history of radiotherapy with development of the sarcoma within or adjacent to the irradiated field; (2) a sufficient latency period between radiation exposure and sarcoma development, with no evidence that the sarcoma was present before irradiation; and (3) histological confirmation that the secondary sarcoma is distinct from the primary malignancy [9]. Murray et al. further proposed defining the irradiated field according to the 5% isodose line when the original radiotherapy dosimetry is available [9].

In the present case, two of these criteria were clearly fulfilled. First, the colonic angiosarcoma was diagnosed 16 years after radiotherapy, representing an appropriate latency period for a radiation-associated sarcoma. Second, the secondary vascular malignancy was histologically distinct from the patient’s previous cervical squamous cell carcinoma. However, the criterion related to the irradiated field could not be definitively established because the original radiotherapy plan, radiation dose, and treatment fields were unavailable. Consequently, it was not possible to confirm that the cecal and right-colonic tumor arose within the irradiated volume or the 5% isodose line. The history of pelvic radiotherapy, the prolonged latency, and the histologically distinct nature of the secondary tumor therefore support a possible radiation-associated etiology, although only a temporal association can be established. Nevertheless, a sporadic primary colonic angiosarcoma cannot be formally excluded.

The clinical presentation of colorectal angiosarcoma is nonspecific and is largely determined by the tumor site and extent. Gastrointestinal bleeding is the most frequently reported manifestation and may present as melena, hematochezia, chronic iron-deficiency anemia, or acute hemorrhage. Other reported symptoms include abdominal or perianal pain, weight loss, altered bowel habits, intestinal obstruction, intraperitoneal bleeding, and manifestations related to metastatic disease [5,7,10]. In the present case, intermittent rectal bleeding, profound anemia, hemorrhagic ascites, and colonic obstruction were consistent with both the vascular nature and the locally advanced presentation of the tumor. The elevated CA-125 level was considered nonspecific and potentially related to the marked ascites and possible peritoneal irritation.

Endoscopic findings are variable and may include an ulcerated or polypoid mass, friable hypervascular mucosa, active bleeding, or a stenosing lesion. These appearances are not specific and may resemble colorectal adenocarcinoma, inflammatory lesions, or other vascular tumors [7,10]. Although histological confirmation is required, endoscopic biopsy may be associated with a risk of bleeding because of the marked vascularity of the lesion. The decision to perform biopsies should therefore consider the severity of active bleeding, the endoscopic appearance, and the availability of endoscopic or surgical hemostatic management. In our patient, colonoscopy revealed an obstructing, ulcerated, and contact-bleeding mass initially suspected to represent colorectal adenocarcinoma. The diagnosis of angiosarcoma was established only after histopathological and immunohistochemical examination of the endoscopic biopsies.

Cross-sectional imaging is essential for defining the extent of the lesion, identifying complications, and assessing metastatic spread, but it generally lacks features specific to angiosarcoma. Reported findings include irregular or circumferential bowel-wall thickening, a hypervascular mass, intraluminal or intraperitoneal bleeding, and distant metastases, most commonly involving the liver and lungs [5,7]. In the present case, computed tomography demonstrated circumferential cecal and right colon thickening with abundant hyperdense ascites, but no active contrast extravasation or focal hepatic or splenic lesions. The hemorrhagic ascites was an important indicator of the vascular and aggressive nature of the tumor, despite negative ascitic-fluid cytology.

Histologically, angiosarcoma displays a broad morphological spectrum that varies with the degree of differentiation. Well-differentiated tumors are characterized by irregular, anastomosing vascular channels lined by atypical endothelial cells, whereas poorly differentiated tumors may show predominantly solid, spindle-cell, or epithelioid growth patterns with limited vasoformation. Nuclear atypia, hyperchromasia, prominent nucleoli, mitotic activity, and an infiltrative growth pattern support the malignant nature of the vascular proliferation [7].

Immunohistochemistry is essential, particularly in poorly differentiated tumors. CD31 is considered one of the most sensitive and relatively specific markers of endothelial differentiation, whereas CD34 expression is less specific and may be variable. Nuclear expression of ETS-related gene (ERG) or Friend leukemia integration 1 (FLI1) provides additional evidence of vascular differentiation, while factor VIII-related antigen is relatively specific but less sensitive, especially in poorly differentiated lesions [7,11]. A panel of markers is therefore preferable to reliance on a single endothelial marker.

The main differential diagnoses include poorly differentiated carcinoma, gastrointestinal stromal tumor, melanoma, and Kaposi sarcoma. Cytokeratin staining helps distinguish angiosarcoma from carcinoma, although cytokeratin expression may occur in epithelioid angiosarcoma. HHV8 negativity argues against Kaposi sarcoma. Depending on the morphology, additional immunohistochemical markers may be required to exclude these differential diagnoses [12,13]. In our patient, the tumor cells were positive for CD31 and CD34 and negative for cytokeratin, CD3, CD20, and HHV8, with a Ki-67 proliferation index of 40%. In conjunction with the vascular morphology, this immunophenotype supported the diagnosis of a poorly differentiated colonic angiosarcoma. Although ERG or FLI1 staining would have provided additional diagnostic support, these markers were not reported in the available pathological assessment. Myelocytoma (MYC) amplification has been described more frequently in radiation-associated and chronic lymphedema-associated angiosarcomas than in primary sporadic angiosarcomas [14]. Molecular assessment of MYC may therefore provide supportive evidence when available. However, MYC testing was not performed in our patient, representing an additional limitation in assessing a possible radiation-associated etiology.

Because colorectal angiosarcoma is exceptionally rare, no treatment guidelines specific to this anatomical site have been established. Current management is extrapolated from retrospective series of angiosarcomas arising at other sites and from the limited number of published gastrointestinal cases [7,10]. For localized and technically resectable disease, complete surgical excision with negative margins remains the preferred treatment and offers the best possibility of prolonged disease control. In colorectal angiosarcoma, surgery may also be required urgently to manage obstruction, perforation, or uncontrolled bleeding. The extent of resection should be determined according to tumor location, local extension, metastatic status, and the patient’s general condition. The role of adjuvant chemotherapy after complete resection remains uncertain. In unresectable, recurrent, or metastatic angiosarcoma, systemic treatment may include taxane-based therapy, particularly weekly paclitaxel, or anthracycline-based chemotherapy. However, evidence regarding their effectiveness in gastrointestinal and radiation-associated colonic angiosarcoma is extremely limited and is derived mainly from retrospective studies and isolated case reports. Treatment should therefore be individualized within a multidisciplinary sarcoma team [7,10]. In the present case, surgical resection was considered because of the obstructing and bleeding colonic mass. However, the patient’s rapidly deteriorating condition and the occurrence of a massive bilateral pulmonary embolism precluded definitive surgical or systemic treatment.

The prognosis of colorectal angiosarcoma is generally poor, although precise survival estimates remain uncertain because the available evidence is based on isolated case reports and small, heterogeneous retrospective series. Published reviews have reported limited long-term survival, with apparently better outcomes among patients who underwent complete surgical resection [8,15-17]. These estimates should be interpreted cautiously because of the limited number of patients, potential publication bias, and differences in tumor stage and treatment. Adverse prognostic factors reported for angiosarcoma include metastatic disease at diagnosis, incomplete or impossible surgical resection, large tumor size, high histological grade, and older age. However, the prognostic value of individual factors has not been consistently demonstrated across studies [8,15-17]. Early diagnosis and complete resection appear to be the main potentially favorable factors in localized colorectal disease. Our patient had several unfavorable clinical features, including severe anemia, hemorrhagic ascites, complete colonic obstruction, and a poorly differentiated tumor with a high proliferative index. Her clinical course was fulminant, with death occurring seven days after admission following a massive bilateral pulmonary embolism, before any tumor-directed treatment could be undertaken.

## Conclusions

Colonic angiosarcoma is an exceptionally rare and aggressive vascular malignancy that should be considered in the differential diagnosis of an ulcerated, hypervascular, or bleeding colorectal mass, particularly in patients with a history of radiotherapy. Diagnosis requires histopathological examination supported by an appropriate immunohistochemical panel. Complete surgical resection remains the preferred treatment for localized and resectable disease, although the overall prognosis is poor.

In the present case, the 16-year latency after pelvic radiotherapy and the histological distinction between the colonic angiosarcoma and the previous cervical carcinoma raised the possibility of a radiation-associated etiology. However, the absence of the original radiotherapy plan prevented definitive confirmation that the tumor arose within the irradiated field. This limitation warrants cautious interpretation of the causal relationship.
